# De Winter syndrome may be an early electrocardiogram pattern of acute myocardial infarction, two cases report

**DOI:** 10.1111/anec.12729

**Published:** 2019-11-24

**Authors:** Lingzhi Zhang, Yuncao Fan, Jinzhong Xu, Jianxin Yan, Qianzi Ruan, Xiaoyan Jiang

**Affiliations:** ^1^ Department of Electrocardiogram The First People's Hospital of Wenling Wenling China; ^2^ Department of Cardiology The first people's Hospital of Wenling Wenling China; ^3^ Department of Clinical pharmacy The first people's Hospital of Wenling Wenling China; ^4^ Department of Clinical laboratory The First People's Hospital of Wenling Wenling China; ^5^ Department of bone and Joint surgery The First People's Hospital of Wenling Wenling China

**Keywords:** acute coronary syndrome, acute myocardial infarction, De Winter syndrome, electrocardiogram

## Abstract

**Background:**

De Winter syndrome is an electrocardiogram (ECG) pattern related to acute occlusion of the anterior descending artery. The incidence rate of De Winter syndrome is rare, but still requires much attention from clinicians.

**Methods:**

Two patients who finnaly diagnosed with De Winter syndrome were included in our study.

**Results:**

A 55‐year‐old male farmer, who was previously healthy, came to the emergency room due to sudden pain in the precordial area for 6 hours, accompanied with back pain and sweating. The second ECG revealed De Winter syndrome. Emergency coronary angiography was taken, which showed a severe atrioventricular block; diffuse stenosis in the proximal and middle segments of the left anterior descending branch, with 90% stenosis in the severest region. Percutaneous coronary intervention (PCI) of the left anterior descending artery was performed. A 70‐year‐old man with a history of hypertension arrived at the Emergency Department with chest pain for 3 hours. The first ECG was performed, which was contacted with de winter syndrome. The second ECG demonstrated acute anterior Myocardial infarction. Emergency coronary angiography showed approximately 95% stenosis at the junction of the proximal and middle segments. PCI of the proximal and middle segments of the left anterior descending artery was performed.

**Conclusion:**

De Winter syndrome is a type of acute coronary syndrome, which may be an early ECG pattern in the development of acute ST‐segment elevation myocardial infarction. Therefore, once De Winter syndrome is observed on the ECG, acute coronary syndrome, especially acute anterior descending occlusion should not be ignored.

**Abbreviations**
ACS: acute coronary syndrome
AMI: acute myocardial infarction
cTnI: cardiac troponin I
ECG: electrocardiogram
ED: emergency department
LAD: left anterior descending artery
PCI: percutaneous coronary intervention

## 1 INTRODUCTION

De Winter syndrome is an electrocardiogram (ECG) pattern related to acute occlusion of the left anterior descending artery (LAD), which was first described by de Winter et al. in 2008. The incidence rate of De Winter syndrome is approximately 2% of all patients with acute anterior myocardial infarction (Winter, Verouden, Wellens, & Wilde, 2008), which is relatively rare, but still requires much attention from clinicians. Here, we report the medical history of two cases of De Winter syndrome as follows:

## 2 MEDICAL HISTORY

### 2.1 CASE 1

**Figure 1:**
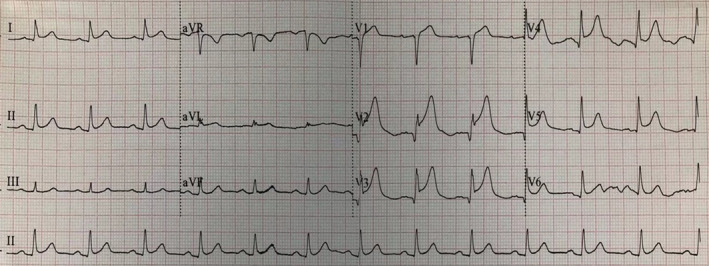
The initial ECG of case 1 patient showed an acute extensive anterior wall myocardial infarction: Abnormal Q waves, combined with an upsloping elevation ST segment of 1.0–3.5 mm and tall, prominent, symmetric T wave appeared in the V1‐V5 leads, a 0.5 mm depression of ST segment was noted in lead avR

**Figure 2:**
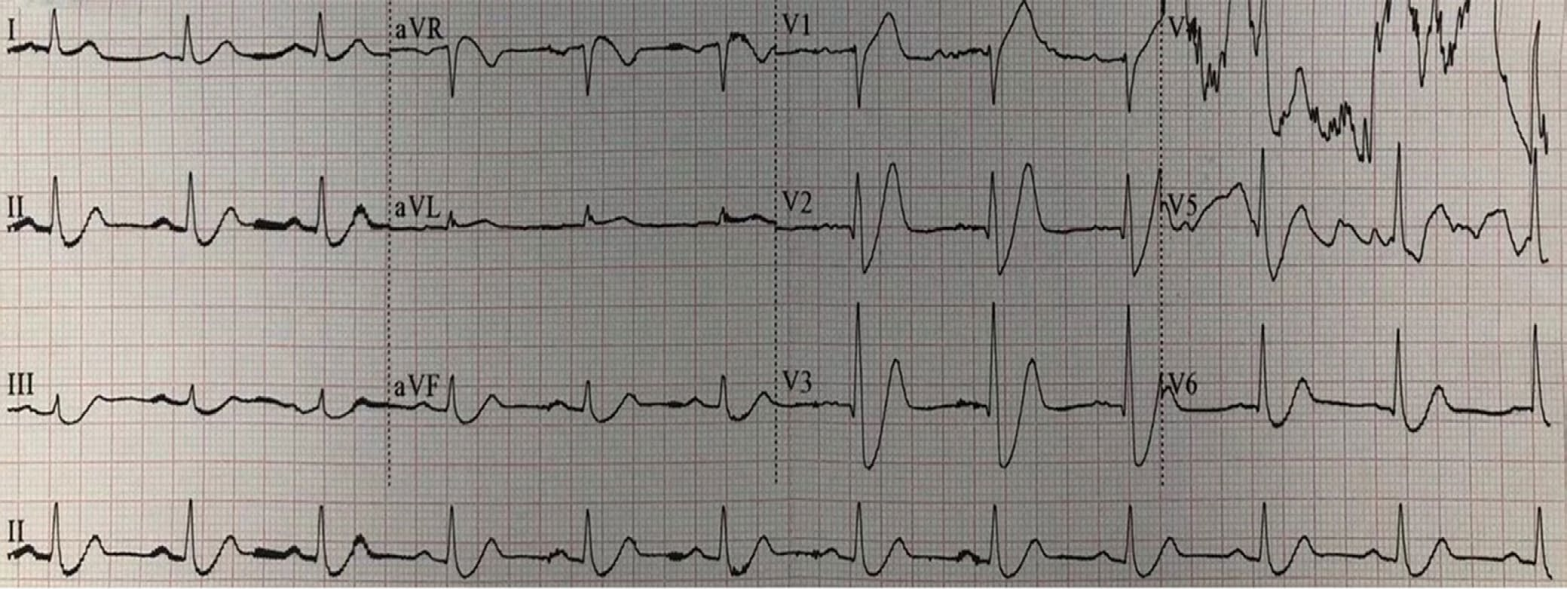
The second ECG of case 1 patient revealed De Winter syndrome: in the precordial leads(V2‐V6) and the inferior leads (II, III, and avF) saw an upsloping ST‐segment depression of 1.5–8 mm at the J point, followed by peaked, symmetric T wave. The ST‐segment elevation of lead avR was about 1.5 mm, and pathological Q waves still could be seen in the leads V2‐V5

**Figure 3:**
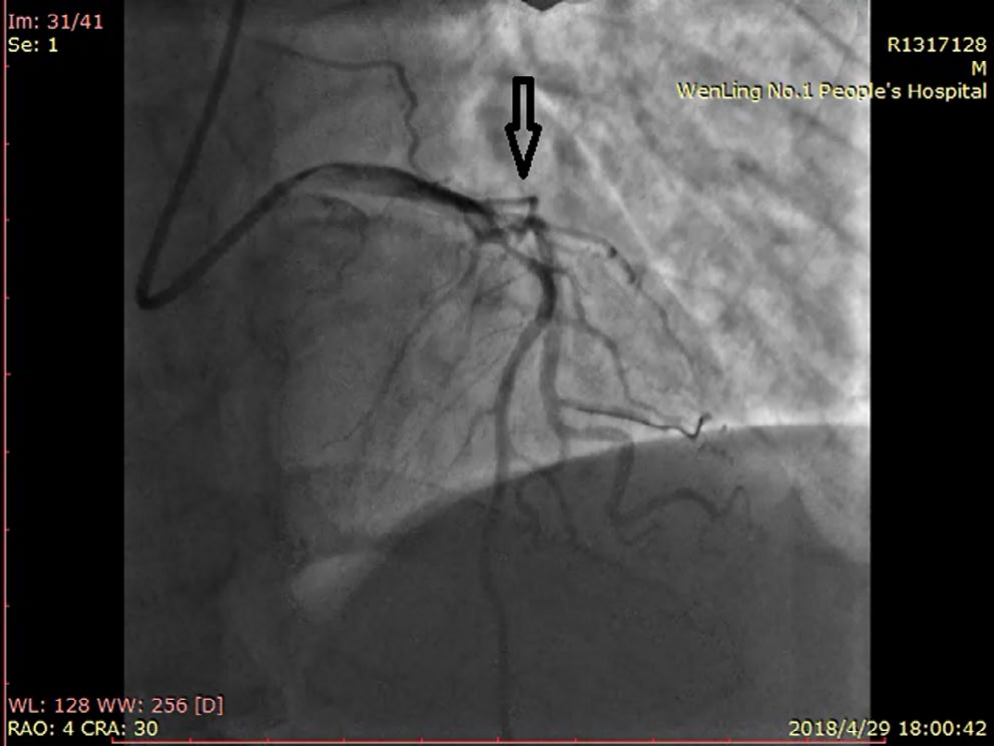
Emergency coronary angiography of case 1 patient showed 90% stenosis in the proximal and middle segments of LAD; and 70% stenosis in the distal segment of LCA

The patient was a 55‐year‐old male farmer who was previously healthy. At 15:50 on April 29, 2018, the patient came to the Emergency Department (ED) of our hospital due to sudden pain in the precordial area for 6 hr. At 15:52, blood sample was collected for an emergency cardiac troponin I (cTnI) test（Beckman ASSECC2）, and at 17:02, the laboratory result showed cTnI level of 0.06 ng/ml (normal reference value of 0.033 ng/ml). The level of cTnI reached 79.27 ng/ml on April 30 and 7.52 ng/ml on May 3. The initial ECG was performed at 16:08 and showed an acute extensive anterior wall myocardial infarction (Figure 1). The patient was given a loading dose of aspirin and clopidogrel (300 mg and 300 mg) for antiplatelet treatment, and morphinye for analgesia. 8 min after the first ECG, the second ECG revealed De Winter syndrome (Figure 2). Emergency coronary angiography demonstrated 90% stenosis in the proximal and middle segments of LAD; and 70% stenosis in the distal segment of the left circumflex artery (LCA) (Figure 3). Percutaneous coronary intervention (PCI) of LAD was performed after consultation with family members.

### 2.2 CASE 2

**Figure 4:**
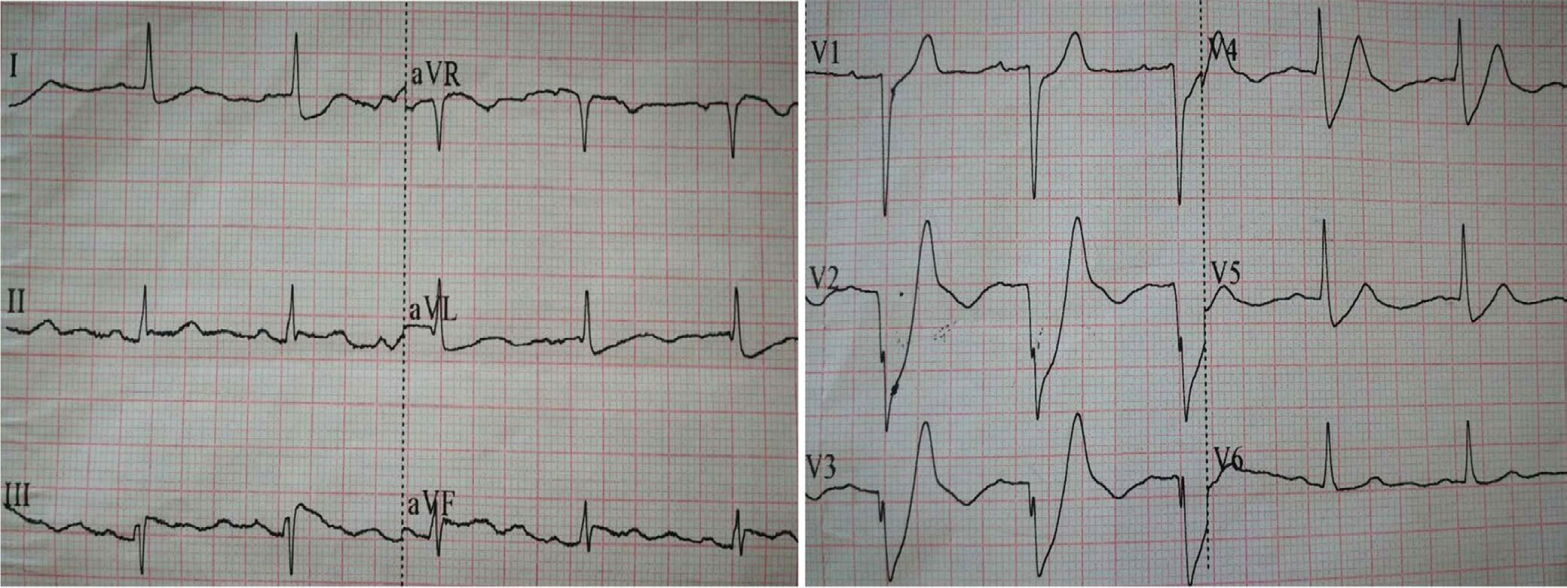
The first ECG of patient of case 2 demonstrated upsloping ST‐segment depression at the J point (1 to 11 mm) in the precordial and high side wall leads (V1‐V5, I and avL) that continued into tall, positive, symmetrical T waves, QRS complex in the V1‐V3 leads were almost QS type. Slight ST‐segment elevation was found in the leads II, III, avF, and avR (approximately 0.5 mm ST‐segment elevation in avR), which was contacted with De Winter syndrome

**Figure 5:**
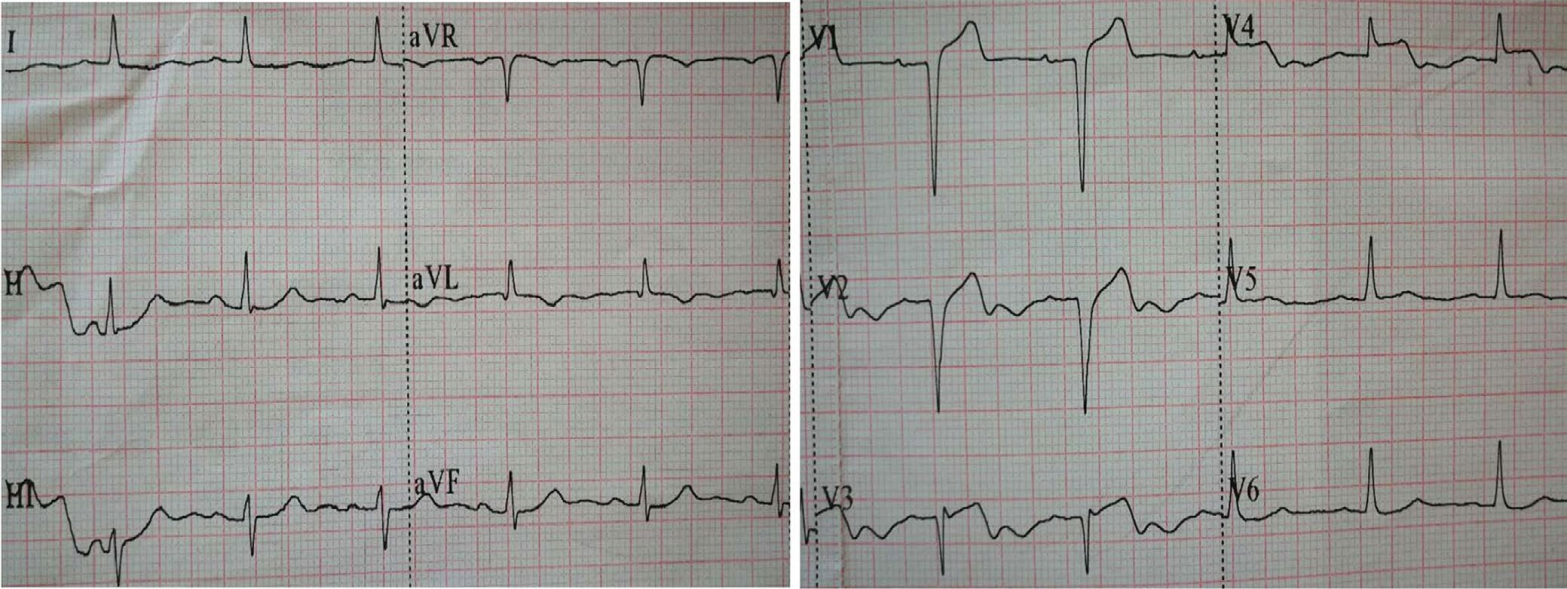
The reviewed ECG of patient of case 2 demonstrated acute anterior myocardial infarction: QS type in the V1‐V3 leads and ST‐segment elevation of 0.5–1.5 mm in the V1‐V4 leads

**Figure 6:**
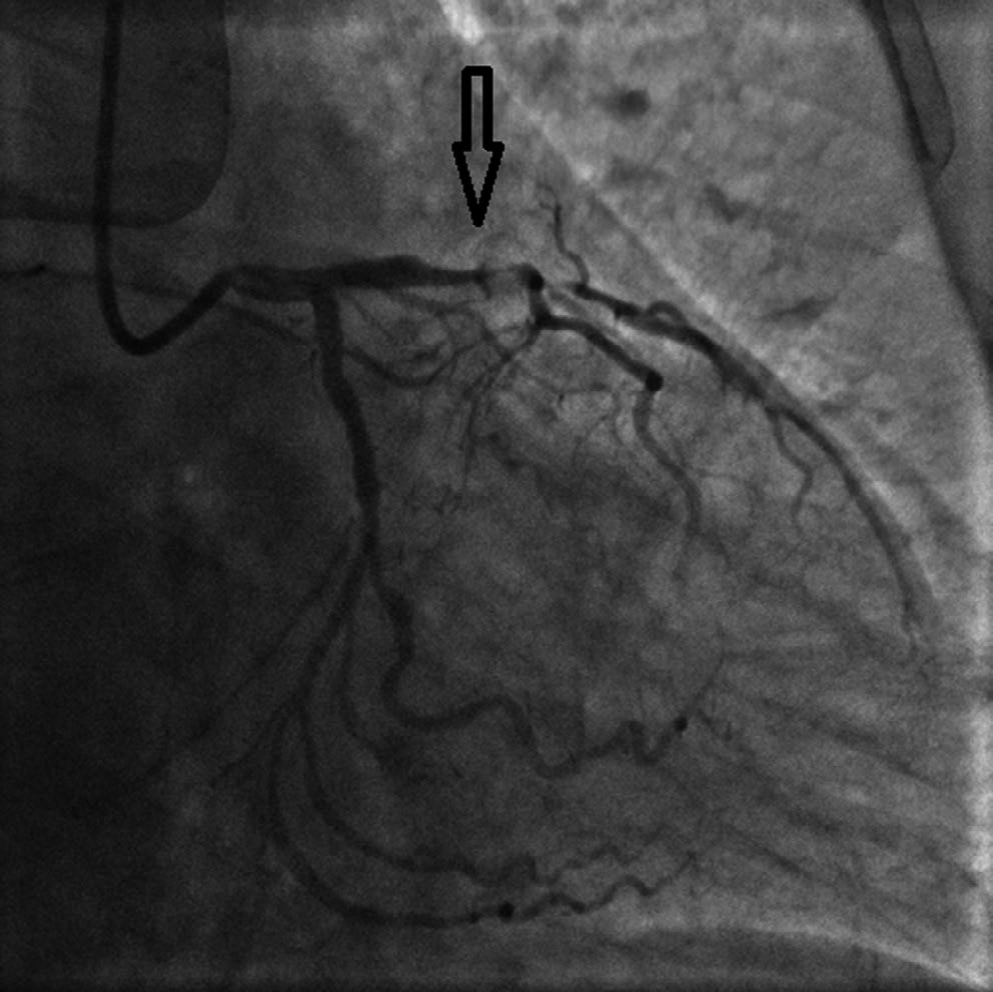
Emergency coronary angiography of case 2 patient showed approximately 95% stenosis at the junction of the proximal and middle segments of LAD, 90% stenosis in the proximal and middle segments of the large diagonal branch

A 70‐year‐old man with a history of hypertension arrived at the ED with chest pain, which had started 3 hr before. The first ECG was performed at 16:15 on May 12, 2018, and demonstrated De Winter syndrome (Figure 4). The cTnI level (blood sample collected at 16:07, and laboratory result obtained at 17:32) was 0.07 ng/ml (normal reference value of 0.033 ng/ml). Acute coronary syndrome (ACS) was considered, and the patient was treated with aspirin (300 mg), Plavix (300 mg), morphine (5 mg), and nitroglycerin (5 mg) via micropump. The reviewed ECG was collected at 17:33 and demonstrated acute anterior myocardial infarction (Figure 5). Emergency coronary angiography showed (Figure 6) approximately 95% stenosis at the junction of the proximal and middle segments of LAD, 90% stenosis in the proximal and middle segments of the large diagonal branch. After PCI, the patient safely returned to the cardiac intensive care.

## 3 DISCUSSION

De Winter syndrome is a type of acute coronary syndrome, and the affected blood vessel is mostly the left anterior descending branch, as previously reported by some scholars (Goktas, Sogut, Yigit, & Kaplan, 2017; Martinez‐Losas & Fernandez‐Jimenez, 2016). However, Jose et al observed the De Winter pattern was related to the occlusion of first diagonal branch arising very proximal from the LAD in a 53‐year‐old male patient (Montero Cabezas et al.,^2016^).

The specific ECG patterns of De Winter syndrome are as follows (Winter et al., 2008): (a) A 1‐to 3‐mm upsloping ST‐segment depression at the J point in leads V1 to V6 that continued into tall, positive symmetrical T waves; (b) QRS complex were usually no widened or only slightly widened; (c) in some patients, there was a loss of precordial R‐wave progression; (d) there was a 1‐to 2‐mm ST‐segment elevation in aVR in most.

Different from the static ECG pattern of De Winter syndrome proposed by De Winter et al (Winter et al., 2008), the above two patients both demonstrated dynamic ST‐segment changes. For both patients in this report, whether the ST segment was elevated first and then depressed, or depressed first and then elevated, the De Winter syndrome changes noted on ECG were found earlier than the laboratory cTnI results, which is not mentioned in the previous literature. In the previous literature, a review of the De Winter syndrome ECG pattern and emergency cTnI examinations showed that when the ECG pattern of De Winter syndrome had been recorded, the troponin I level was still largely normal or only slightly increased (Table 1). Laboratory testing for emergency cTnI requires a process of blood sample collection, separation, and analysis, which usually spends a lot of time. In contrast, in the detection of acute myocardial infarction (AMI), ECG is more convenient, intuitive, and can be repeated in a short time. The relationship between De Winter syndrome and emergency cTnI which was mentioned above should prove that De Winter syndrome may be an early ECG pattern of AMI (Xu, Wang, Liu, & Chen, 2018). When the De Winter pattern is recorded, the myocardium is still in the ischemic phase and has not progressed to complete myocardial infarction or is the result of spontaneous reperfusion after myocardial infarction (Morris & Body, 2017).

**Table 1:** The reported time of the emergency cTnI examination in the previous literature on De Winter syndrome, and the corresponding reference values provided

Literature	Emergency cTnI examination	Reference value	The time of ECG recording of De Winter syndrome	Report time of the cTnI value
Raymond Pranata et al (Pranata & Huang, 2018)	Negative	N/A	On arrival ED	N/A
Mathew Goebel et al (Goebel et al., 2014)	Normal	N/A	On arrival ED	N/A
Fuad Samadov et al (Samadov et al., 2014)	28 ng/L (High‐sensitivity troponin I)	0–14 ng/L	4:30 p.m.	7:30 p.m.
Kai‐Chun Hu et al (Hu et al., 2019)	0.06 ng/ml	0.5 ng/ml	On arrival ED	An hr later
Rex Pui Kin Lam et al (Lam et al., 2019)	Not performed	N/A	28 min after arrival ED	N/A
Case 1	0.06 ng/ml	0.033 ng/ml	16:08	17:02
Case 2	0.07 ng/ml	0.033 ng/ml	16:15	17:32

In summary, De Winter syndrome is a type of ACS, which may be an early ECG pattern in the development of AMI. Therefor, once De Winter syndrome is observed on the ECG, acute coronary syndrome, especially acute left anterior descending occlusion should not be ignored.

## CONFLICT OF INTEREST

None declared.

## AUTHORS' CONTRIBUTIONS

Zhang LZ and Fan YC designed the study and collected data. Zhang LZ, Xu JZ, and Ruan QZ prepared tables and figures. Zhang LZ, Yan JX, and Jiang XY reviewed the results, and Zhang LZ wrote the manuscript. All authors have made an intellectual contribution to the manuscript and approved the submission.
